# ELMOD3-Rab1A-Flotillin2 cascade regulates lumen formation via vesicle trafficking in *Ciona* notochord

**DOI:** 10.1098/rsob.220367

**Published:** 2023-03-15

**Authors:** Amei Liu, Xiuke Ouyang, Zhuqing Wang, Bo Dong

**Affiliations:** ^1^ Fang Zongxi Center, MoE Key Laboratory of Marine Genetics and Breeding, College of Marine Life Sciences, Ocean University of China, Qingdao 266003, People's Republic of China; ^2^ Institute of Evolution & Marine Biodiversity, Ocean University of China, Qingdao 266003, People's Republic of China; ^3^ Laoshan Laboratory, Qingdao 266237, People's Republic of China

**Keywords:** notochord lumen, vesicle trafficking, ELMOD3, flotillin2, Rab1A

## Abstract

Lumen development is a crucial phase in tubulogenesis, although its molecular mechanisms are largely unknown. In this study, we discovered an ELMO domain-containing 3 (ELMOD3), which belongs to ADP-ribosylation factor GTPase-activating protein family, was necessary to form the notochord lumen in *Ciona* larvae*.* We demonstrated that ELMOD3 interacted with lipid raft protein Flotillin2 and regulated its subcellular localization. The loss-of-function of Flotillin2 prevented notochord lumen formation. Furthermore, we found that ELMOD3 also interacted with Rab1A, which is the regulatory GTPase for vesicle trafficking and located at the notochord cell surface. Rab1A mutations arrested the lumen formation, phenocopying the loss-of-function of ELMOD3 and Flotillin2. Our findings further suggested that Rab1A interactions influenced Flotillin2 localization. We thus identified a unique pathway in which ELMOD3 interacted with Rab1A, which controlled the Flotillin2-mediated vesicle trafficking from cytoplasm to apical membrane, required for *Ciona* notochord lumen formation.

## Graphical abstract

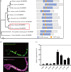

## 1. Introduction

The ADP-ribosylation factor (ARF) family of GTPases is the key regulator of various biological processes such as directional membrane trafficking (secretion and endocytosis), ciliogenesis, cytoskeleton remodelling, apoptosis and lipid metabolism [1,2]. ELMO (cell engulfment and motility) domain-containing proteins (ELMODs) have recently been identified as novel ARF GTPase-activating proteins (GAPs) with highly conserved arginine residues, but no common GAP domain [3]. ELMODs belong to the ELMO family proteins that share a common ELMO domain and consist of six members named ELMOs (ELMO1-ELMO3) and ELMODs (ELMOD1-ELMOD3) based on their size, structure and function [3,4]. The ELMODs are ancient proteins in diverse eukaryotes. ELMOs are predicted to originate from the ELMODs [3,5,6]. ELMO domain is a common feature linking ELMOs and ELMODs proteins. The ELMOs interact with dedicator of cytokinesis proteins and play as an unconventional GEFs of Rac GTPases to regulate cytoskeleton rearrangement, polarity establishment, cell migration and cell apoptosis [3,4,7,8]. While ELMOD1-ELMOD3 exhibit the GAP activity for ARFs and ARLs to regulate protein trafficking, membrane remodelling, ciliogenesis and lipid metabolism [5,6,9,10].

In different biological models, ELMODs bind with broad substrates including ARFs and ARLs [6,11,12]. In *Arabidopsis thaliana*, the loss-of-function of ELMOD-A and ELMOD-E influences the number, position and shape of apertures [5]. ELMOD2 is required for meiosis progression in mouse oocytes through controlling mitochondrial dynamics. ELMOD2 is important in regulating mitochondrial fusion, cytokinesis, ciliogenesis, microtubule stability and lipid metabolism at lipid droplets in mouse embryonic fibroblasts [6,9,13]. ELMOD1 influences the secretory pathway and lipid droplets in HeLa cells, and the overexpression of ELMOD1 alters Golgi morphology involving ARFs [9]. The loss-of-function of ELMOD1 or ELMOD3 leads to the disrupted vesicle trafficking from the Golgi to cilia. ELMOD1 and ELMOD3 act at the Golgi and cilia to regulate ciliogenesis and ciliary protein traffic linking ARL3 or ARL16 [6,10]. All preceding evidence implies that ELMODs regulate vesicle trafficking as a GAP via binding with ARF or ARL small GTPases.

Tubulogenesis is an essential step in the development of tubular organs such as the lungs, blood arteries and gut [14]. Lumen formation is a critical step in tubulogenesis that involves vesicle trafficking [15–17]. Lumen development requires continuous vesicle transport and timely replenishment to increase the membrane area. Secretory trafficking and vesicle fusion are conveyed across the apical membranes during high-velocity intracellular vesicle trafficking, demonstrating that vesicle trafficking is responsible for the extension of the apical membrane domain [18]. Previous studies have shown that the vesicle uptake could be associated with RhoA [19,20], Rac [21], Rab [22], Arf [23,24], caveolae and Flotillin2 [25,26]. It requires a constant balance between endocytosis and exocytosis. The precise coordination and cooperation in the process are unknown.

Notochord cells in ascidian *Ciona* species form luminal structures during embryonic development [16,27]. As we found that in this study, ELMOD3 was highly expressed and enriched in the ascidian notochord apical cell membrane. The loss-of-function of ELMOD3 resulted in no visible lumen in the *Ciona* notochord, indicating that ELMOD3 is indispensable for lumen formation. Furthermore, ELMOD3 interacted with Flotillin2 and Rab1A, and regulated Flotillin2-mediated vesicle trafficking via Rab1A. Our findings identified a novel signalling mechanism that regulated the formation and expansion of tubular lumens during tubulogenesis.

## 2. Results

### 2.1. Evolutionarily conserved ELMODs in *Ciona*

**Figure 1.**
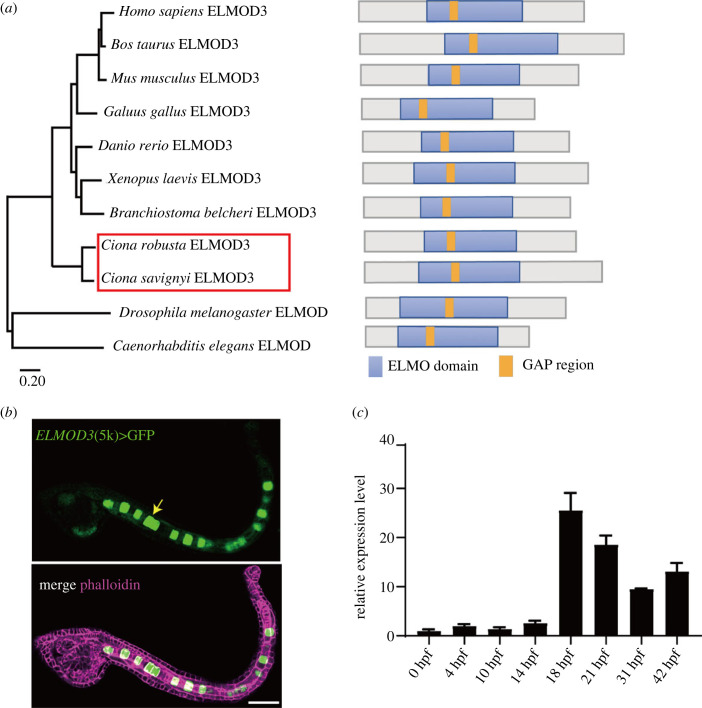
Structure and expression pattern of *Ciona-ELMOD3.* (*a*) Phylogenetic analysis and domain composition of ELMOD3 in different species. Phylogenetic analysis of ELMOD3 using the maximum likelihood by MEGA11. ELMO domain (blue) and GAP region (orange) were presented in ELMOD3 in diverse species. (*b*) Mosaic expression pattern of a 5 kb upstream genome region of *ELMOD3* fused with GFP (*ELMOD3*(5 kb)>GFP). The white arrowhead indicated that the GFP signal was observed in notochord cells. Scale bar, 50 µm. (*c*) The mRNA expression level of *ELMOD3* at different development stages in *Ciona* embryos and larvae.

ELMOD3 was recognized in the *Ciona* genome by Blastp, and domain analysis revealed the existence of an ELMO domain and a GAP region based on multiple sequence alignment in various species (figure 1*a*; electronic supplementary material, figure S1). A phylogenetic tree built using the full-length protein sequence revealed that ELMOD3 was highly conserved among chordates (figure 1*a*). The upstream 5000 bps of *Ciona-ELMOD3* was fused with fluorescent protein GFP to make a construct that was electroporated into *Ciona* fertilized eggs to detect tissue expression patterns. Results revealed that GFP signalling was primarily expressed in *Ciona* notochord cells (figure 1*b*). Furthermore, we performed qRT-polymerase chain reaction (PCR) analysis of *Ciona-ELMOD3* using different developmental-staged embryos. The findings revealed that *ELMOD3* had a comparatively high expression level during 18 and 21 h post fertilization (hpf), when the lumen was developed and expanded (figure 1*c*). These findings suggest that *Ciona-*ELMOD3 plays a vital role in notochord tubulogenesis.

### 2.2. ELMOD3 is required for *Ciona* notochord lumen formation

**Figure 2.**
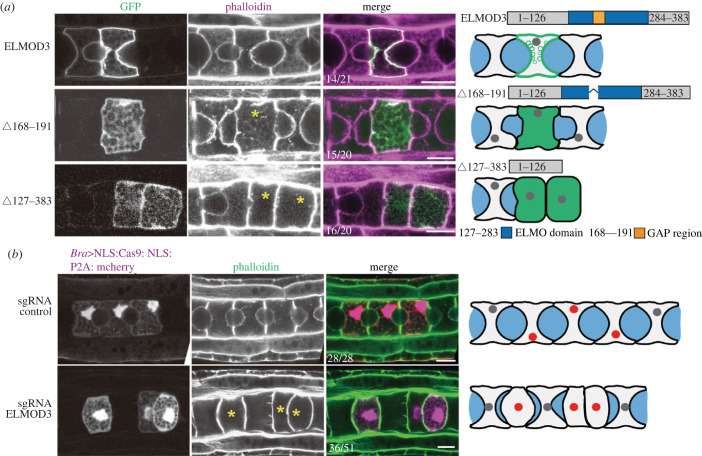
ELMOD3 is required for notochord lumen formation. (*a*) The expression of *Ciona*-ELMOD3 DN mutants prevented notochord lumen formation. The asterisk represents defects on notochord lumen expansion. The ratios of embryos to phenotypes are labelled. The schematic images on the right side of the confocal images described the phenotypes of the *Ciona*-ELMOD3 DN mutants and control. (*b*) Confocal images of *Ciona-ELMOD3* CRISPR mosaic embryos and control ones at the late tailbud stage. The asterisk represents defects on notochord lumen expansion. The ratio of embryos with phenotypes was labelled. The schematic images on the right side of the confocal images described the phenotypes of the *Ciona-ELMOD3* knockdown/out and control. The light grey, grey, blue and red areas represented the notochord cells, the nuclei, lumen and the nuclei in cells expressed Bra > NLS:Cas9:NLS:P2A:mCherry (red), respectively. The scale bar represents 10 µm.

We produced two ELMOD3 mutants with GAP active region deletion (Δ168–191) or C-terminal deletion (Δ127–383), which serve as dominant negative (DN) to reveal the roles of ELMOD3 in *Ciona* notochord tubulogenesis [3,9]. Two DN constructs were forced to be expressed in notochord cells using notochord-specific promoters. The findings revealed abnormalities in lumen development in mutant-expressing cells (yellow asterisk cell in figure 2*a*), indicating that ELMOD3 is required for notochord lumen formation.

We further knocked out *ELMOD3* using the CRISPR/Cas9 approach to validate its function in lumen formation. The expression level of ELMOD3 protein was dramatically reduced in the CRISPR-ELMOD3-KO group compared to the control group, based on western blotting results (electronic supplementary material, figure S2A and B). Considering the mosaic expression pattern of ELMOD3 during *Ciona* embryogenesis, the considerable reduction in ELMOD3 protein level demonstrated that the sgRNA ELMOD3 employed in CRISPR/Cas9 knockout tests was efficient. We discovered a failure of lumen creation in mCherry-expressing notochord cells due to the co-expression of sgRNA ELMOD3 and Cas9-mCherry, showing that the loss-of-function of ELMOD3 halted lumen formation (figure 2*b*). Overall, these findings suggest that ELMOD3 is required for the formation of notochord lumen.

### 2.3. Flotillin2 interacts with ELMOD3, regulating notochord lumen formation and expansion

**Figure 3.**
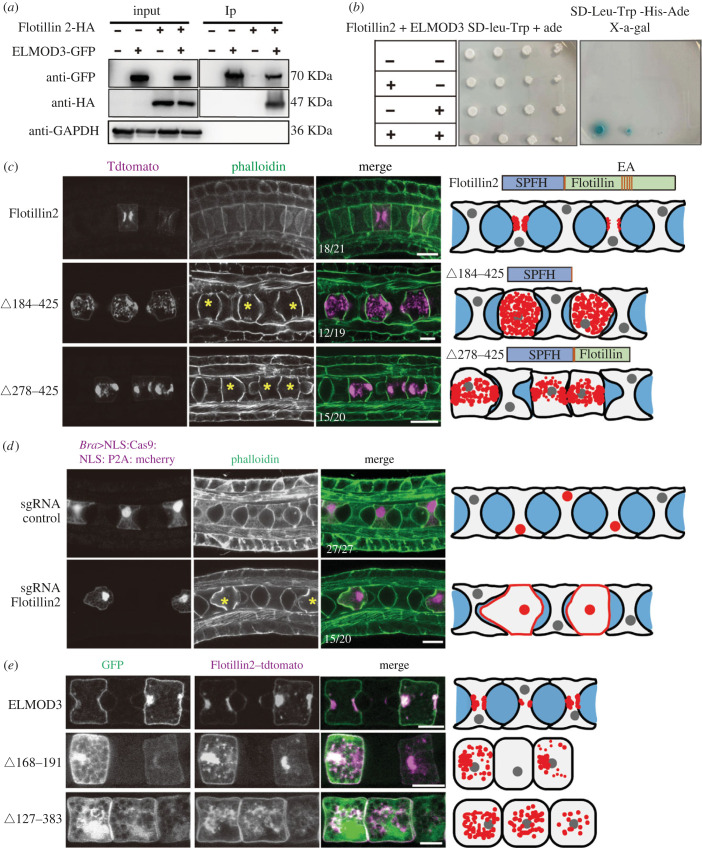
Apical membrane localized Flotillin2 is required for notochord lumen formation. (*a,b*) ELMOD3 bound with Flotillin2. ELMOD3 interacted with Flotillin2 by Co-IP assay in HEK-293T cells and Y2H assay. (*c*) The overexpression of Flotillin2 mutants prevented lumen formation. Left panel: the Flotillin2 wild-type or mutant construct was overexpressed in notochord cells, respectively. Yellow asterisks indicate the notochord cell overexpressing Flotillin2 wild-type or mutants. Right panel: schematic representation of the phenotype of mutant overexpression. (*d*) The knockout of Flotillin2 suppressed lumen formation. Left panel: confocal images of Flotillin2-KO and control group embryos at 21 hpf, respectively. Yellow asterisks indicate the notochord cell of Flotillin2-KO. Right panel: schematic images depict the phenotypes of the Flotillin2-KO. (*e*) The apical localization of Flotillin2 depends on ELMOD3. Left panel: the co-expression of ELMOD3 wild-type or mutants and Flotillin2 in notochord cells and the localization of Flotillin2 is abnormal in these notochord cells overexpressing ELMOD3 mutants compared with control. Right panel: schematic diagrams depicting Flotillin2 localization. The grey, red and blue areas represent the nuclei, Flotillin2 and lumen, respectively. Scale bars, 10 µm.

With an immunoprecipitation assay with proteins expressed in cultured tumour cells, we investigated molecular mechanism, by which ELMOD3 regulates lumen formation. Flotillin2, produced in notochord cells, was identified as one of 243 putative interaction proteins (electronic supplementary material, figure S3). Using co-immunoprecipitation (Co-IP) and yeast two-hybrid (Y2H) experiments, we validated the interaction between ELMOD3 and Flotillin2 (figure 3*a,b*). Furthermore, we demonstrated that the N-terminal region of ELMOD3 interacted with the SPFH domain of Flotillin2 (electronic supplementary material, figure S4) [28,29].

We developed mutants of flotillin2 (Δ184–425) and flotillin2 (Δ278–425), acting as DN to validate if *Flotillin2* is involved in the development of the notochord lumen [30] (figure 3*c*). *Ciona* has no discernible lumen after overexpressing Flotillin2 mutants. We subsequently knocked out *Flotillin2* using the CRISPR/Cas9 method to corroborate the findings. The effectiveness of the sgRNA *Flotillin2* was demonstrated by western blotting results that showed the expression level of the Flotillin2 protein was much lower in the CRISPR-*Flotillin2*-KO group compared to the control (electronic supplementary material, figure S2C and D). We co-expressed Cas9-mCherry and sgRNA *Flotillin2*, and the results revealed that lumen formation failed in mCherry-expressing notochord cells, demonstrating that the loss-of-function of Flotillin2 halted lumen formation (figure 3*d*).

In *Ciona* notochord cells, flotillin2 was distributed at the apical membrane and co-localized with ELMOD3 (figure 3*e*). The fact that Flotillin2's membrane localization signalling sequence was not predicted, however, suggests that Flotillin2's membrane localization is dependent on recruitment from other partners. Therefore, we hypothesized that ELMOD3 might control how Flotillin2 is distributed across membranes. To accomplish this, we examined the distribution of Flotillin2 in *Ciona* notochord cells overexpressing ELMOD3 mutants. The findings demonstrated that Flotillin2 lost its apical membrane localization in cells expressing the ELMOD3 mutant (figure 3*e*), demonstrating that Flotillin2's apical localization was ELMOD3 dependent.

### 2.4. Rab1A regulates the localization and trafficking of Flotillin2 in the notochord cells

**Figure 4.**
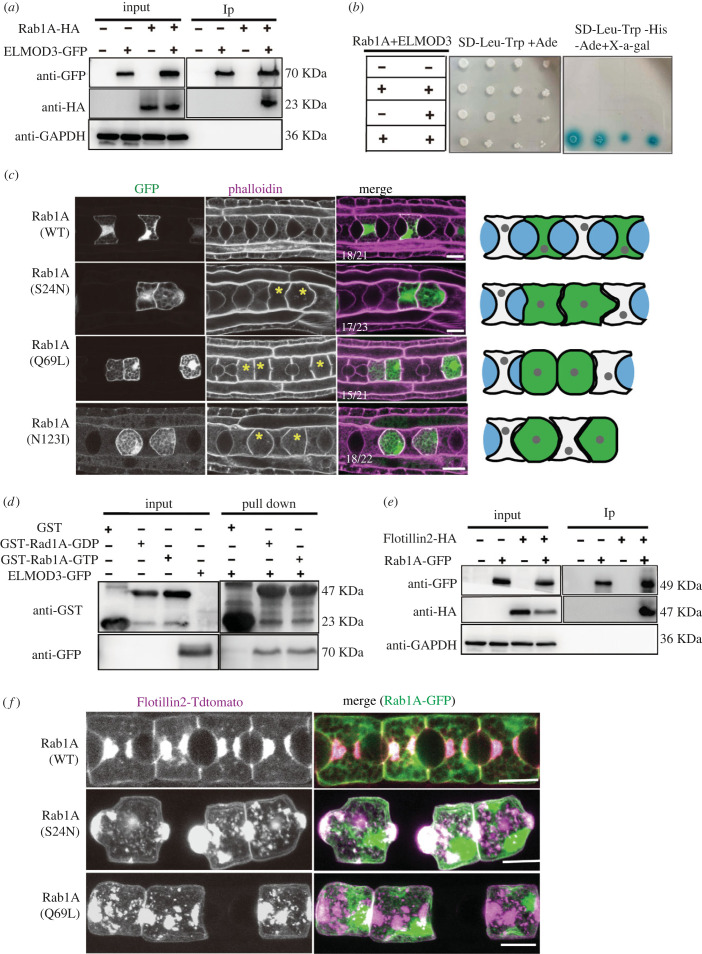
Rab1A regulates the localization and trafficking of Flotillin2 in the notochord cells. (*a,b*) Interaction between ELMOD3 and Rab1A verified by immunoblot analysis (with anti-GFP and anti-HA) in HEK-293T cells (*a*) and yeast two-hybrid experiments (*b*). (*c*) The expression of Rab1A mutants with notochord-specific promoter prevented lumen formation. Left panel: confocal images of Rab1A mutants and control group embryos at 21 hpf stages, respectively. Yellow asterisks mark the notochord cells. The statistical embryo number is shown as a white number. Right panel: schematic representation depicting the phenotype of the Rab1A mutants. (*d*) ELMOD3 interacted with Rab1A by GST pull-down assay. Immobilized GST and GST-Rab1a were converted into GDP (inactive) and GTP (active) forms, respectively, then loaded with the extract of HEK-293T cell-expressing ELMOD3-GFP. The bound protein complex was detected by anti-GFP antibody. (*e*) Interaction between Flotillin2 and Rab1A using Co-IP assay in HEK-293T cells. (*f*) The localization of Flotillin2 was altered in Rab1A mutations. Left panel: the co-expression of Rab1A wild-type or mutants with Flotillin2 in notochord cells. Scale bars, 10 µm.

The list of possible proteins that could interact with ELMOD3 included Rab1A. Using Co-IP and Y2H assays, we further verified the relationship between ELMOD3 and Rab1A (figure 4*a,b*). Additionally, we discovered that Rab1A interacted with N-terminal region of ELMOD3 (electronic supplementary material, figure S5A and B). Using pure recombinant GDP or GTP*γ*S, a pull-down experiment was conducted to ascertain whether ELMOD3 serves as a GAP of Rab1A [31,32]. The result showed that ELMOD3 bound directly with Rab1A GTP and Rab1A GDP form (figure 4*d*), suggesting that ELMOD3 is Rab1A target not a GAP.

Using the upstream driver experiment, we proved that *Rab1A* was expressed in notochord cells to determine if it was involved in the development of the notochord lumen (electronic supplementary material, figure S3). Then, we developed the constitutively active mutant Rab1A(Q69L) and the Rab1A DN constructs Rab1A(N123I) and Rab1A (S24N) [33]. The expression of Rab1A mutants in the notochord cells failed in visible lumen formation, the bulging membrane at the region where the lumen was expected to form in the notochord (figure 4*c*), phenocopying the loss-of-function of ELMOD3 and Flotillin2. These results indicate that Rab1A is required for notochord lumen formation.

The interaction between Rab1A and ELMOD3 suggests that Rab1A might be involved in regulating Flotillin2. We confirmed that Rab1A directly interacted with Flotillin2 (figure 4*e*) using Co-IP experiments. We co-expressed Rab1A mutants Rab1A(S24N) and Rab1A(Q69L), and Flotillin2 in the notochord cells to examine the roles of Rab1A on Flotillin2 vesicle trafficking. The results showed that the localization of Flotillin2 was disrupted in the notochord cells (figure 4*f*), verifying that Rab1A plays a role in the localization of Flotillin2.

**Figure 5.**
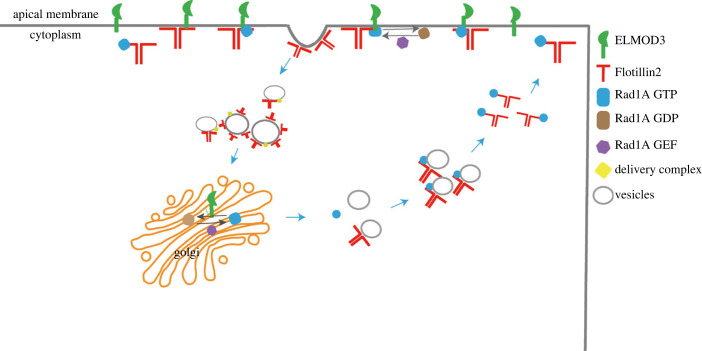
Working model of ELMOD3-Rab1A as a signalling cascade on regulating the Flotillin vesicle trafficking that is essential for lumen formation and expansion in *Ciona* notochord. Flotillin2 was transported to the apical membrane by Rab1A and targeted to the apical membrane through the transmembrane structure of ELMOD3. Flotillin2 induced the membrane to bend and to achieve endocytosis. Simultaneously, Rab1A was inactivated by ELMOD3 and left Flotillin2. Flotillin2 wrapped the vesicles and transported them to the corresponding position. Subsequently, in the cytoplasm, Rab1A transported Flotillin2 to the plasma membrane. The molecular signal pathway of ELMOD3-Rab1A-Flotillin2 was established to regulate lumen formation.

Overall, ELMOD3 interacted with Rab1A that controls Flotillin2 disassembly in the apical endocytic complex, facilitating Flotillin2 turnover (figure 5). As a result, we establish a novel ELMOD3-Rab1A-Flotillin2 cascade that contributes to notochord lumen formation and expansion.

## 3. Discussion

### 3.1. ELMOD3 is required for lumen formation in *Ciona*

Lumen development is a crucial step in tubulogenesis. It necessitates the interplay of numerous biological processes, including cell polarity establishment, vesicle trafficking (exocytosis and endocytosis) and dynamic cytoskeleton remodelling [18,34]. During the polarity establishment process, the Par3-Par6-aPKC polarity complex is transported to the middle of the anterior and posterior lateral membrane region, where the lumen will appear (the initiation of lumen formation), that is essential for AB polarity and lumen formation in many models [18,27,35]. The absence or mis-localization of the Par3-Par6-aPKC complex has been shown to impede the development of a lumen in *Ciona* notochord [36]. The centrosome regulates polarity complex localization in *Caenorhabditis elegans* and *Drosophila* system models [37,38]. Previous research suggested that proteins from the ELMO family might be involved in controlling this process [39]. In the present study, we observed the polarized localization of ELMOD3 and the formation of vesicle structure on the apical membrane in notochord cells, which indicates that ELMOD3 might play a role in the establishment of the cell polarity process.

After AB polarity was established, vesical trafficking was required for lumen development and expansion in lumenogensis. IRSp53 deficiency disrupted vesicle trafficking to the apical membrane, resulting in aberrant lumen formation in epithelial tubules [40]. In *C. elegans*, exocyst-targeted vesicles are delivered to the luminal membrane and induce lumen expansion [41]. Blocking the vesicle trafficking by treatment with BFA (Brefeldin A), an inhibitor that inhibits trafficking from the endoplasmic reticulum to the Golgi apparatus, inhibited lumen formation in *Ciona* [26]. In this study, the knockout of *ELMOD3* arrested the notochord lumen formation. ELMODs (ELMOD1-3), as novel Arf GAP member, lack the canonical Arf GAP domain, but have a conservative GAP active region in ELMO domain. Subtle mutations of GAP active region are usually sufficient to reduce GAP activity [42]. A previous work has shown that overexpressed the ELMOD2 mutant of N-terminal non-GAP region blocked the trafficking of adipocyte triglyceride lipase to lipid droplets LDs and membranes [9]. It is also possible the mutant proteins could disrupt interactions with the binding partners of the endogenous proteins. The sequences of GAP active regions of ELMOD3 and ELMOD2 are highly consistent, suggesting similar mutation of ELMOD3 could also serve as a DN one to interfere with the endogenous protein. Our findings reveal that deleting the GAP region caused aberrant lumen formation, implying that GAP activity is required for the ELMOD3 function. Previous research indicated that ELMOD3 functioned as an ARF GAP [6]. It exhibited GAP activity on ARL2/3 involved in vesicle trafficking and membrane remodelling [6,43]. Based on our findings and earlier research, we hypothesized that ELMOD3 regulates notochord lumen development via vesicle trafficking.

### 3.2. The apical membrane localization of Flotillin2 depends on ELMOD3

The protein–lipid rafts Flotillin is involved in various cellular processes, including cell migration and adhesion, endocytosis, cytoskeletal remodelling and cell signalling events [44]. Many pieces of research have demonstrated that Flotillin1 and Flotillin2 mediate a clathrin-independent endocytosis pathway [45,46]. Flotillin-1 knockdown hindered clathrin-independent cholera toxin absorption and endocytosis of a GPI-linked protein in mammalian cells [47]. In zebrafish, Flotillins have been shown to be required for cholera toxin trafficking and toxicity [48]. Flotillins consist of an N-terminal SPFH domain forming membrane microdomains and a C-terminal flotillin domain. The expression of mutation of the C-terminal flotillin domain could serve as a DN to form homo- and hetero-oligomers with the endogenous one [49,50], which is crucial for Flotillin-mediated membrane curvature and endocytosis [46,47,50]. Similarly, we produced mutants of Flotillin2 as DN in *Ciona* notochord. In this investigation, we discovered that knocking down Flotillin2 hindered lumen formation, mimicking the loss-of-function of ELMOD3. We found that ELMOD3 interacted with Flotillin2's SPFH domain. Loss-of-ELMOD3 resulted in Flotillin2's mis-localization in notochord cells and the failure of lumen formation and expansion. These findings suggest that ELMOD3 regulates apical membrane localization of Flotillin2 via its SPFH domain and that Flotillin2 distribution and traffic are required for notochord lumen development.

### 3.3. ELMOD3 acts as a novel target of Rab1A that required for the localization of Flotillin2

Rab proteins are members of the Ras-like small GTPase superfamily, and they regulate intracellular membrane trafficking by taking part in numerous transport processes like as exocytosis and endocytosis [51,52]. In the *Drosophila* trachea, the early endosome localization protein Rab5 is in charge of luminal protein clearance from the lumen to epithelial cells [53]. Rab9 is involved in recycling the luminal protein Serpentine, which is necessary for regulating tube shape in the *Drosophila* trachea [32]. Recycling apical membrane proteins mediated by Rab11A is critical for developing a single lumen in the zebrafish gut [14].

Rab1A has been shown to be involved in vesicular protein transport from the ER to the Golgi as well as cell adhesion and migration [54]. In this investigation, we discovered Rab1A not only in the intracellular but also in the cell membrane, implying that it plays a role in vesicle trafficking from the Golgi to the cell membrane. Rab1A mutations halted lumen development in notochord cells, mimicking the loss-of-function of ELMOD3. We found that ELMOD3 interacted with Rab1A GTP and Rab1A GDP form, respectively. Meanwhile, we discovered that Flotillin2 was mislocalized in Rab1A mutant notochord cells. Previous research has shown that Integrin1 lipid raft localization is dependent on Rab1A [54]. Our findings imply that Rab1A is responsible for the location of the lipid raft protein Flotillin2 in *Ciona* notochord cells and that ELMOD3, as a Rab1A target, regulates Flotillin2 disassembly in the apical membrane, which is essential for *Ciona* notochord lumen formation.

## 4. Methods

### 4.1. Ascidians and embryos

Adult ascidians were collected from Qingdao and Rongcheng (Shandong Province, China). The animals were kept in the Sars-Fang-Center ascidian culture facility. Eggs were then dissected and mixed in seawater with sperm from other individuals. The embryos were grown at 16°C after fertilization and electroporation.

### 4.2. Plasmid construction

For the promoter study, the pEGFP-N1 vector was subcloned with the 5 kb DNA sequence upstream of the ELMOD3 gene and the 3 kb DNA sequences upstream of the *Flotillin2* and *Rab1A* genes from *Ciona*, which were amplified by PCR.

Full-length cDNA of *Ciona*-*ELMOD3*, *Flotillin2* and *Rab1A* were amplified by PCR. These PCR products were cloned into *Brachyury >* EGFP-N1 or *Brachyury* > tdtomato-N1 vectors, generating the *Brachyury* > ELMOD3-EGFP, *Brachyury* > Flotilllin2-tdtomato and *Brachyury* > Rab1A-GFP expression plasmids. These PCR products were cloned into *CMV* > GFP and *CMV* > HA vectors, generating the *CMV* > ELMOD3-GFP, *CMV* > Rab1A-GFP*, CMV* > Flotillin2-HA and *CMV* > Rab1A-HA for cell experiments. The full-length of *Rab1A* PCR products was cloned into the PGEX-6p-GST vector to express the *RAB1A* protein.

Flotillin2 and Rab1A, the N-terminal and C-terminal deletion mutants of *ELMOD3* and *Flotillin2* from *Ciona,* were amplified and subcloned into *Brachyury* > EGFP vector, then ligated by infusion reaction to create *Brachyury* > ELMOD3(Δ168–191)-EGFP, *Brachyury* > ELMOD3(Δ127–283)-EGFP, *Brachyury* > Flotillin2(Δ184–425) and *Brachyury* > Flotillin2(Δ278–425) expression plasmids to generate mutants of ELMOD3. Point mutations such as *Brachyury* > Rab1A(S24N)-EGFP, *Brachyury* > Rab1A(Q69L)-EGFP and *Brachyury* > Rab1A(N123I)-EGFP expression plasmids were generated by PCR-based site-directed mutagenesis (site-directed mutagenesis kit, Vazyme, Nanjing, China).

PCR amplified the ELMOD3 and then ligated with prey bait vector pGBKT7 to generate pGBKT7-ELMOD3, according to the manufacturer's instructions to construct pGADT7*-*Flotillin2, pGADT7*-*Rab1A. Using the same strategy, the other constructs: pGBKT7-ELMOD3 (Δ1–283), pGBKT7-ELMOD3 (Δ127–283), pGBKT7-ELMOD3 (Δ192–383), pGBKT7-ELMOD3 (Δ1–191), pGBKT7-ELMOD3 (168–191), pGADT7-Flotillin2 (Δ184–425) and pGADT7*-*Flotillin2 (Δ278–425) for yeast two-hybrid assay were also generated. All constructs were generated using the ClonExpress II one-step cloning kit (Vazyme, Nanjing, China) and confirmed by sequencing. All the PCR primers are listed in the electronic supplementary material, table S1.

### 4.3. Quantitative PCR

Total RNA was extracted with RNAiso plus (TAKARA, Japan) from different stages (0, 4, 10, 14, 18, 21, 31 and 42 hpf) of *Ciona* embryos/larvae. All cDNAs were synthesized using 1 µg total RNA by HiScript II Q RT SuperMix for qPCR (Vazyme, Nanjing, China). qPCR amplification was performed using the SYBR Mix kit (Vazyme, Nanjing, China) using a Light Cycler 96 (Roche, Basel, Switzerland). The reaction condition was as follows: 95°C for 30 s, 40 cycles at 95°C for 10 s and 60°C for 30 s, 95°C for 15 s, 60°C for 60 s, and 95°C for 15 s. Tubulin was used as the reference gene. Data were calculated using the 2^−ΔΔCt^ method and the graphs of qPCR results were made by Prism 9 software. Details of the primers used are listed in the electronic supplementary material, table S1.

### 4.4. Yeast two-hybrid assay

As previously described, the two-hybrid yeast screen was conducted using the Matchmaker GAL4-based two-hybrid system (Clontech) [55]. *ELMOD3* prey vectors with *Flotillin2* or *Rab1A* bait vectors in different combinations were co-transformed into yeast strain AH109. The transformed cells were selected on a double dropout medium (SD/-Leu/-Trp). They compared with positive controls (SD/-Ade/His/-Leu/-Trp) and X-α-gal groups and cultured at 30°C for 3–6 days. In parallel, the combination of pGBKT7, pGBKT7 and pGADT7 vectors was used as negative controls.

### 4.5. Electroporation

Electroporation was performed according to the previously described method with some modifications [26]. After fertilization, the dechorionated eggs (300 µl) were mixed with 30–40 µg of plasmids and were made up to 100 µl final volume using MilliQ water. The prepared mix was electroporated with 420 µl of 0.96 M d-Mannitol using a Gene Pulser X cell system (BIO-RAD) in 4 mm cuvettes. As a parameter, the exponential protocol was used with 50 V and 1500/2000 µF. After electroporation, embryos were allowed to develop to the desired stages at 16°C for confocal observation.

### 4.6. Immunohistochemistry

Embryos were fixed with 4% paraformaldehyde in seawater for 2 h at room temperature. After fixation, the embryos were washed three times (once 20 min) with PBS containing 0.1% Triton X-100 (PBST). Alexa phalloidin 488 nm or Alexa phalloidin (1/100 dilution) 555 nm was used overnight at 4°C. The embryos were washed three times for 8 h at room temperature and then mounted on glass slides with DAPI for confocal microscopic observation.

### 4.7. Cell culture, transfection and co-immunoprecipitation

HEK-293T cells were cultured in Dulbecco's modified Eagle's medium (Invitrogen, USA) supplemented with fetal bovine serum (10%, Gibco, USA), penicillin (100 µg ml^−1^) and streptomycin (100 mg ml^−1^, Sangon Biotech), under conditions of 5% CO_2_ at 37°C. Cells were subcultured for further experiments after they became 80%–90% confluent. The transfection was performed according to the manufacturer's instructions using Lipofectamine 3000 Invitrogen (Thermo Fisher).

After transfection (48 h), the cells were collected and lysed in buffer containing 50 mM Tris–HCl (pH8), 75 mM NaCl, 1 mM MgCl2, 0.05% NP-40, 100 mM sucrose, 1 mM DTT and 1xProtease Cocktail inhibitors (Roche) for 30 min at 4°C. Lysates were centrifuged for 10 min at 14 000 rpm to remove debris. The supernatant was incubated with GFP-tap beads (gta 20, chromotek, Germany) and incubated on a rotator overnight at 4°C. The immunoprecipitants were washed with 1 ml wash-buffer containing 10 mM Tris–HCl (pH7.5), 150 mM NaCl, 0.5 Mm EDTA, 1 mM DTT and 1xProtease inhibitors Cocktail (Roche) for three times. The immunoprecipitants were eluted using 2× SDS-PAGE Sample Buffer and boiled for 10 min at 95°C. Samples were loaded into 10% SDS-PAGE gels and electrophoretically separated.

### 4.8. CRISPR/Cas9

Two target sequences of CRISPR/Cas9 against *ELMOD3* and *Flotillin2* were designed by CRISPRdi-rect (http://crispr.dbcls.jp). The sequences of selected guide RNAs (sgRNAs) and control sgRNA are listed in electronic supplementary material, table S2. Based on the target sequences of CRISPR/Cas9, all sgRNAs were synthesized and cloned into the Cr-U6 > sgRNA(F + E) vector (Addgene number: 59 986) for expressing the sgRNA. The electroporation mixes were as described previously with some modifications [56]. The CRISPR/Cas9 system contained the PCR product of sgRNA (control group) or the mix PCR products of the targeted gene sgRNA (knockout group) (50 µl), plasmid of *Cs-brachyury*(3k) > NLS: Cas9:: NLS:: P2A:: mCherry (40 µg), embryos (300 µl) and electroporation buffer (420 µl). It was then electroporated into fertilized eggs at the following conditions: capacitance: 2000 µF/50 V, and time constant: within the range of 15–20 ms.

### 4.9. GST-fusion proteins production

The GST-Rab1A fusion protein was produced in bacteria using *E. coli bl21 Rosetta (DE3)* competent cells transformed with the pGEX-6P1 vector in which the desired construct had been cloned. pGEX-GST-Rab1A and pGEX-GST were expressed in Rosetta (DE3) competent cells. When grown overnight at 37°C, it reached approximately OD = 0.4–0.6, and protein expression was induced with 1 mM IPTG for 20 h at 18°C. After the induction, the cells were pelleted down at 8000 rpm for 30 min at 4°C. The pellets were used immediately or stored at −80°C.

### 4.10. GST-Rab1A and ELMOD3 *in vitro* binding

GST-Rab1A recombinant proteins were purified by glutathione-sepharose 4B beads (Amersham). GST and GST-Rab1A proteins were immobilized on glutathione-sepharose 4B beads and loaded with GDP or GTP*γ*S as described earlier [32,57]. CMV-*ELMOD3*-GFP was expressed in HEK-293T cells for 48 h. The cells were collected and lysed in buffer containing 50 mM Tris–HCl (pH8), 75 mM NaCl, 1 mM MgCl_2_ and 0.05% NP-40, 100 mM sucrose, 1 mM DTT, 1xprotease Cocktail inhibitors (Roche) for 30 min at 4°C. Lysates were centrifuged, and the supernatant was incubated with GST and GDP- or GTP*γ*S-loaded GST and GST-Rab1A for 2 h at 4°C. The samples were subsequently washed five times with lysis buffer, eluted using SDS sample buffer and analysed by SDS-PAGE.

### 4.11. Western blot analysis

The proteins were separated using 10% SDS-polyacrylamide gel electrophoresis at 200 V for 1.5 h, and then 110 mA for 3 h at 4°C. Protein bands from the gel were transferred onto polyvinylidene fluoride (PVDF) membranes using transfer buffer. The PVDF was blocked with 5% fat-free powdered milk in a blocking solution, including tris-buffered and tween 20 (TBST), at room temperature for 2 h. After washing three times with TBST, the PVDF was incubated overnight at 4°C with primary antibody (Cell Signaling Technology, USA). After incubation, PVDF was washed with TBST and incubated with the secondary antibody (TransGen Biotech, China) at RT for 2 h. The proteins of PVDF were detected using enhanced chemiluminescence (Advansta, Menlo Park, CA, USA). Grey scale analysis was performed using ImageJ, and statistical data analyses were performed by Prism 9 software.

## Data Availability

The datasets supporting the conclusions of this article are included within the article and its additional files. Additional data are provided in the electronic supplementary material [58].
